# Placement of oppositely charged aminoacids at a polypeptide termini determines the voltage-controlled braking of polymer transport through nanometer-scale pores

**DOI:** 10.1038/srep10419

**Published:** 2015-06-01

**Authors:** Alina Asandei, Mauro Chinappi, Jong-kook Lee, Chang Ho Seo, Loredana Mereuta, Yoonkyung Park, Tudor Luchian

**Affiliations:** 1 Department of Interdisciplinary Research, Alexandru I. Cuza University, Iasi, Romania; 2 Center for Life Nano Science, Istituto Italiano di Tecnologia, Rome, Italy; 3 Research Center for Proteineous Materials, Chosun University, Gwangju, South Korea; 4 Department of Bioinformatics, Kongju National University, Kongju, South Korea; 5 Department of Physics, Alexandru I. Cuza University, Iasi, Romania

## Abstract

Protein and solid-state nanometer-scale pores are being developed for the detection, analysis, and manipulation of single molecules. In the simplest embodiment, the entry of a molecule into a nanopore causes a reduction in the latter’s ionic conductance. The ionic current blockade depth and residence time have been shown to provide detailed information on the size, adsorbed charge, and other properties of molecules. Here we describe the use of the nanopore formed by *Staphylococcus aureus* α-hemolysin and polypeptides with oppositely charged segments at the *N*- and *C*-termini to increase both the polypeptide capture rate and mean residence time of them in the pore, regardless of the polarity of the applied electrostatic potential. The technique provides the means to improve the signal to noise of single molecule nanopore-based measurements.

Since its inception over 20 years ago, nanopore-based analysis has evolved as a high-precision single-molecule technology for the study of biomolecular interactions and the sensitive detection of analytes. The measurement principle is simple. Single molecules enter the pore and reduce the ionic current that otherwise flows freely. The concentration, identity, and other physical properties of the molecule (e.g., size, charge, etc.) are inferred from the degree of current reduction and the residence time distributions of the molecule in the pore^1^,^2^,^3^.

Nanopore-based measurements differ substantially from conventional microscopic resistive pulse technology, because without interactions between the analyte and pore, the residence times would be orders of magnitude too short to even detect the molecules, let alone characterize them^3^,^4^. Nevertheless, because some analytes interact with the pore walls, protein- and solid state-based nanopore sensors have emerged as powerful tools for the investigation of various chemistries^3^,^5^,^6^,^7^,^8^,^9^,^10^ or biomolecule detection and analysis (e.g, RNA and DNA^11^,^12^,^13^,^14^,^15^, peptides^16^,^17^,^18^,^19^,^20^,^21^,^22^ proteins^23^,^24^,^25^ and bacterial toxins^26^,^27^).

These applications stimulated the development of a number of theoretical and numerical approaches^28^,^29^,^30^,^31^,^32^ to describe various stages of the process, from analyte capture^33^,^34^ to translocation through the pore^4^,^11^,^12^ and to analyze the factors that account for the magnitude and the duration of the current blockades^35^,^36^.

As described above, one of the major obstacles for making nanopore-based analysis mainstream is the relatively short residence time of analytes in the pore^10^,^11^,^12^. To date, methods to alter the mean analyte residence time in the pore include changing the temperature^37^,^38^, viscosity and electrolyte concentration of the aqueous solution^35^,^38^,^39^, altering the nanopore size and material^40^, rapid switching or modulating the transmembrane potential^41^, adding large macromolecules to one side of analyte^33^, using a processivity enzyme^42^, controlling the balance between the electrostatic and electro-osmotic forces^21^,^43^, employing a pressure-voltage biased pore^44^, controlling surface charge in solid-state nanopores with low-power visible light^45^, using Li^+^ as counterions in the electrolyte solution^46^, coating nanopores with a fluid lipid bilayer^47^, or using optical tweezers^48^.

Here, we describe an approach to control analyte residence times in a single nanopore. The proof-of-principle strategy resorted to using polypeptides whose C- and N-termini contained distinct patches of basic and acidic amino acid segments. The method also provides an increase in the rate of analyte capture by the pore, regardless of the applied potential polarity. This strategy might prove useful for determining the identity of a wide range of biological polymers.

## Results

**Figure 1:**
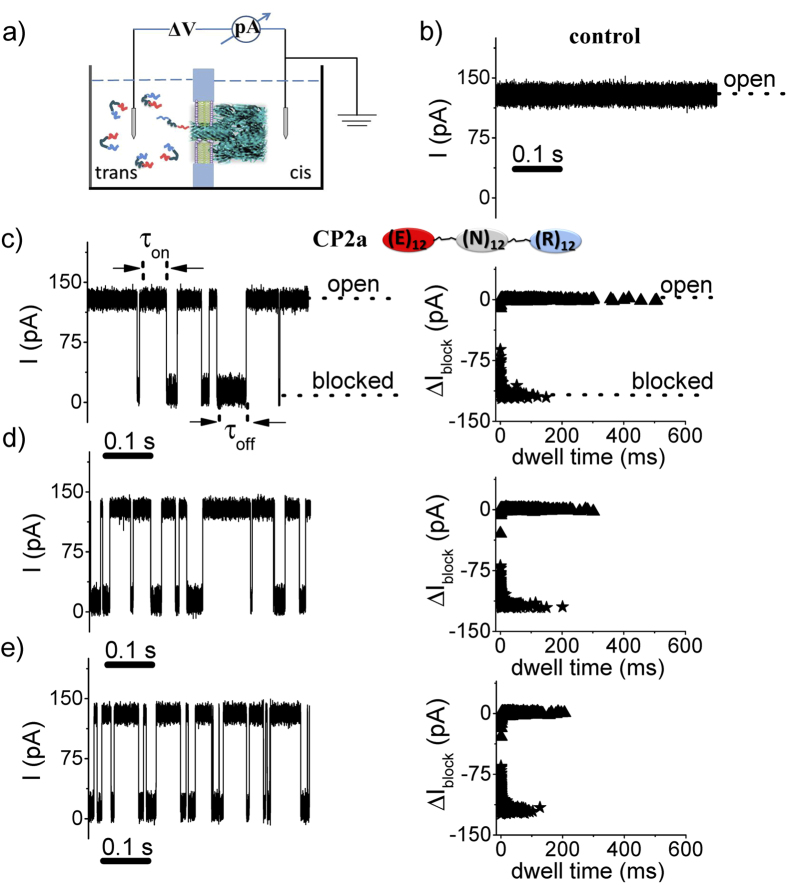
Detecting single polypeptides with a nanopore. **a)** Schematic illustration of the apparatus (note that α-HL, peptides and chamber are not drawn to scale). The pore is formed by and the voltage is applied across the pore via a matched pair of Ag-AgCl electrodes. **b)** Ionic current time series of the open pore. Addition of the CP2a peptide at **c)** 5 μM, **d)** 10 μM, or **e)** 20 μM to the *trans* side causes randomly occurring transient blockades in the current. The applied potential was +70 mV (a positive voltage drives cations from the *trans* to the *cis* compartment) and the solution contained an aqueous electrolyte solution of 2 M KCl, 10 mM HEPES, at pH = 7.3.

Individual molecules of an engineered polypeptide CP2a ((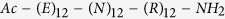)) were detected using single protein nanopores formed in planar lipid bilayers by *Staphyloccocus aureus* α-hemolysin (α-HL)^49^ added to the *cis* chamber (see Fig. 1a). The ionic current through the fully open pore is relatively large (~ 120 pA) and quiescent (see Fig. 1b), and the addition of 5 μM of the engineered polypeptide CP2a to the *trans* side causes transient current blockades which occur at random intervals (see Fig. 1c). Increasing the polypeptide concentration causes the capture rate to increase, as is shown qualitatively in Fig. 1c–e. The current blockades represent the capture of single polypeptides and possibly their translocation through the nanopore^11^.

**Figure 2:**
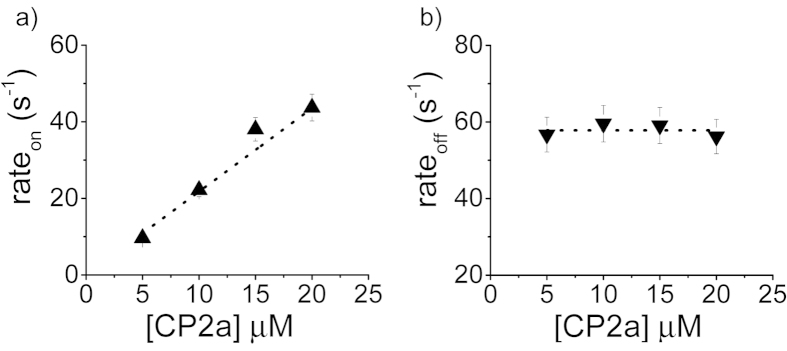
Kinetics of polypeptide:nanopore interactions. The mean capture rate of polypeptide by the pore (*left*) and dissociation rate of the polymers from the pore (*right*) as a function of polypeptide concentration.

The capture rate increases linearly with polypeptide concentration (see Fig. 2a), consistent with the capture of other molecules with the α-HL pore^11^. In contrast, the dissociation rate which is the inverse of mean residence time of the molecules in the pore, estimated from the lifetime probability density distribution, is independent of the polymer concentration (see Fig. 2b). These results suggest a simple bimolecular interaction mechanism between the polypeptide and the pore.

**Figure 3:**
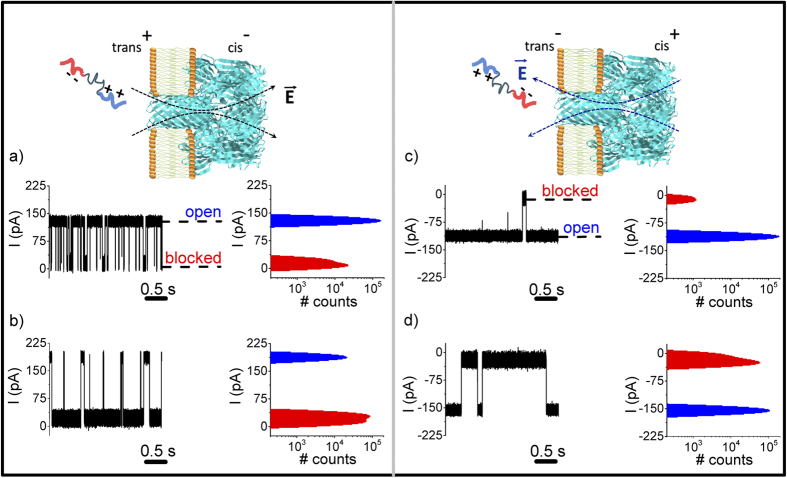
The effect of the applied potential magnitude and polarity on polypeptide-nanopore interactions. The ionic current time series and all-points histograms of the currents for **a**) ΔV = + 70 mV, **b**) ΔV = + 100 mV, **c**) ΔV = − 70 mV, and **d**) ΔV = − 100 mV. As reflected qualitatively by the amplitude histograms, the blockade probability characterizing the peptide-induced reversible obstructions of the ion current through the pore is voltage-dependent (see text).

The polypeptide-induced current blockades are observed at both positive and negative applied potentials (see Fig. 3). This was not the case for negatively charged polynucleotides^11^,^33^, which suggests that the positively and negatively charged ends of the polypetide might account for its ability to partition into the pore regardless of the voltage polarity. Control experiments with another peptide construct (termed CP2), which has a net charge of ~ + 6 e^−^ at both N- and C-termini at neutral pH, showed that this polypeptide is captured by the pore only for positive potentials (see Supplementary Information Fig. S1).

For the polypeptide with negative and positive charge groups at the N and C termini, respectively, the capture rate and residence time of the polymer in the pore depend on the magnitude and polarity of the applied potential (see Fig. 3). Specifically, the capture rate of CP2a is greater for *trans*-positive potentials than for *trans*-negative ones (shown qualitatively in Fig. 3a,c). This asymmetric behavior is likely due to net negative charge located at the *trans* pore entrance caused by 14 aspartic acids (D127 and D128) and 7 lysines (K131)^49^. In 2 M KCl solution (Debye length κ^−1^ ~1.9 Å), this fixed charge distribution will act as an electrostatic energy barrier for polypeptides that try to enter with the negative end first.

Increasing the applied potential magnitude increases the polypeptide residence times, as can be seen qualitatively by comparing Fig. 3a,b (positive potentials) and 3c to 3d (negative potentials). As reflected qualitatively by the amplitude histograms (see Fig. 3), the blockade probability (
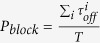
 , where 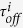 are individual blockade durations seen during the total observation time T) characterizing the peptide-induced reversible obstructions of the ion current through the pore is voltage-dependent: P_block_ = 12 ± 1.5% (ΔV = + 70 mV), P_block_ = 88 ± 1.0% (ΔV = + 100 mV), P_block_ = 1.03 ± 0.53% (ΔV = −70 mV) and P_block_ = 65 ± 0.15% (ΔV = −100 mV).

**Figure 4:**
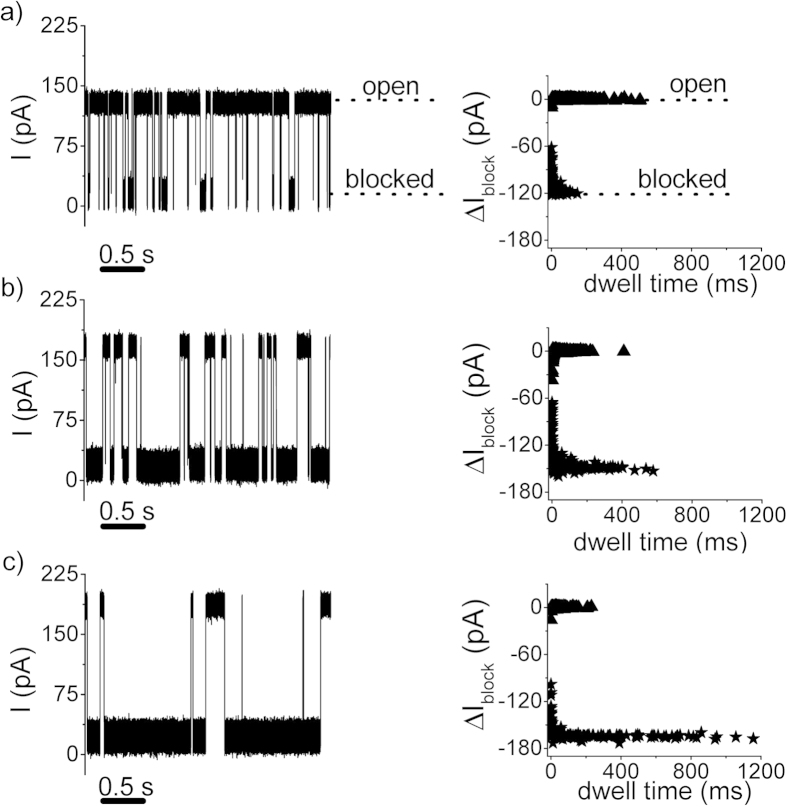
Selected traces showing ion current fluctuations through an open α-HL pore, due to reversible pore blockades by CP2a polypeptides added to the *trans* chamber at a bulk concentration of 5 μM, measured at ΔV = + 70 mV (panel a), ΔV = + 90 mV (panel **b**) and ΔV = + 100 mV (panel **c**). The panels next to the ion current traces display the scatter plot distribution of current blockade vs. inter-events and blockade-events durations associated to the peptide-α-HL interactions, showing that the increase in the holding potential leads concomitantly to **a** decrease of the inter-events time intervals (τ_on_; ‘open’) and an increase of the blockade-events durations (τ_off_; ‘blocked’).

The voltage-dependence of the reversible α-HL conductance blockades by the CP2a peptide provides the landmark observation of this work (*vide supra*), and for concreteness was studied herein in further details at positive transmembrane potentials alone. That is, while the duration of blockade inter-events intervals decreases with the increase in the positively applied transmembrane potential, as it is expected due to the larger electric forces and subsequent increased peptide transport towards the vicinity of the pore, it was striking to note that the residence time of the peptide within the α-HL pore dramatically increases at such greater potentials (see Fig. 4). Similar results are obtained with negative transmembrane potentials (see Supplementary Information Fig. S2).

**Figure 5:**
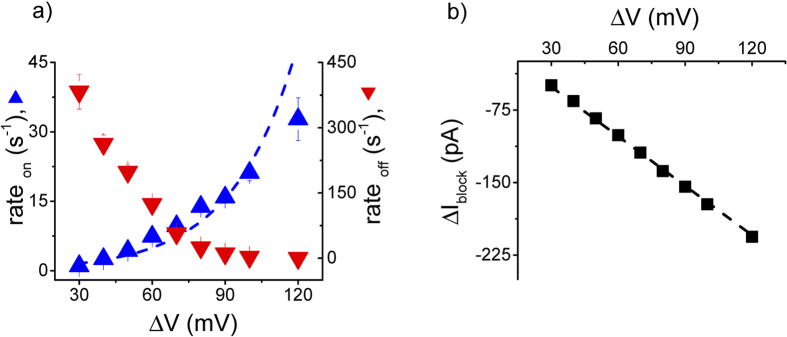
Voltage dependence of the capture rate (rate_on_) and dissociation rate (rate_off_) (panel **a**), and blockade depth (ΔI_block_) (panel **b**) of CP2a polypeptide interacting reversibly with a single α-HL pore. The capture rate was fit to a single exponential 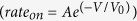 , where A = 0.49 ± 0.12 s^−1^ and V_0_ = − 26.13 ± 2.34 mV.

The distributions of inter-events and blockade-events durations of data shown in Fig. 4 were found exponential (Supplementary Information Fig. S3), and the statistical analysis allowed us to quantify the voltage-dependence of the association (rate_on_) and dissociation (rate_off_) rates of the CP2a-α-HL interactions (see Fig. 5).

The capture rate of polynucleotides by the α-HL pore was shown to increase approximately exponentially with applied potential^33^,^34^. We applied the same van’T Hoff expression to the polypeptide capture rate data (see Fig. 5a). Interestingly, unlike polynucleotides, which required applied potentials in excess of 60 mV to enter the pore, the CP2a polypeptide does so at potentials as low as 30 mV. In addition, when adjusted for the difference in bulk polymer concentration, the prefactor in the exponential for the CP2a polypeptide is ~ 100-fold greater than for polynucleotides^33^, but the value of |V_0_| is approximately the same (26 mV vs. 18 mV). Because the polypeptides are more likely to enter the pore (much greater pre-factor and no threshold potential required to inject the polymer in the pore), the polypeptide is likely more flexible than polynucleotides that have an approximately uniformly negatively charged phosphate backbone.

The dissociation rate of the CP2a polypeptide decreases monotonically with the increase of the applied potential (see Fig. 5a). This is in stark contrast to the voltage dependent residence times for current blockades of the α-HL nanopore caused by negatively charged polynucleotides^11^ and poly(ethylene glycol) molecules that weakly bind monovalent cations^35^,^50^.

Assuming the polypeptide reduces the ionic current solely via volume exclusion and under simplifying assumptions with respect to pore’s topology^25^,^35^,^47^, the modulus of the relative current blockade (ΔI_block _= I_blocked_−I_open_) relates to the volume of the polypeptide that is inside the pore by 
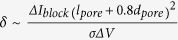
 , where σ is the electrolyte solution bulk conductivity, l_pore_ and d_pore_ are the pore length and diameter, respectively). Figure. 5b shows that the relative current blockade increases proportionally with the applied potential, which suggests the polypeptide conformation in the pore is not altered by the applied electric field, presumably because the polymer is already fully stretched. Thus, the voltage dependence of the capture rate and dissociation rate (see Fig. 5a) are not due to different peptide conformations inside the pore. The polypeptide likely acts as an unstructured polymer chain that moves into the pore with little or no folded intermediates^11^, regardless the applied voltage. Because the mean residence time increases with the magnitude of the applied potential (see Fig. 5a), and thereby increases the signal to noise ratio of the measurement, the use of greater potentials provides an opportunity to further explore the nature of polypeptide-induced conductance changes with the α-HL pore. We therefore measured the current blockades caused another polypeptide (CP2b) that is similar to CP2a, except that the central segment contains 12 glutamines instead of 12 asparagines. One would expect that CP2b would block the pore conductance more than would CP2a because it the volume of a single hydrated glutamine is greater than that of asparagine (~ 142 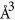 vs. **~** 116 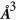), and experiments confirm this (in modulus, (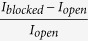) = 0.97 for CP2b vs. 0.92 for CP2a; Supplementary Information Figure S4).

**Figure 6:**
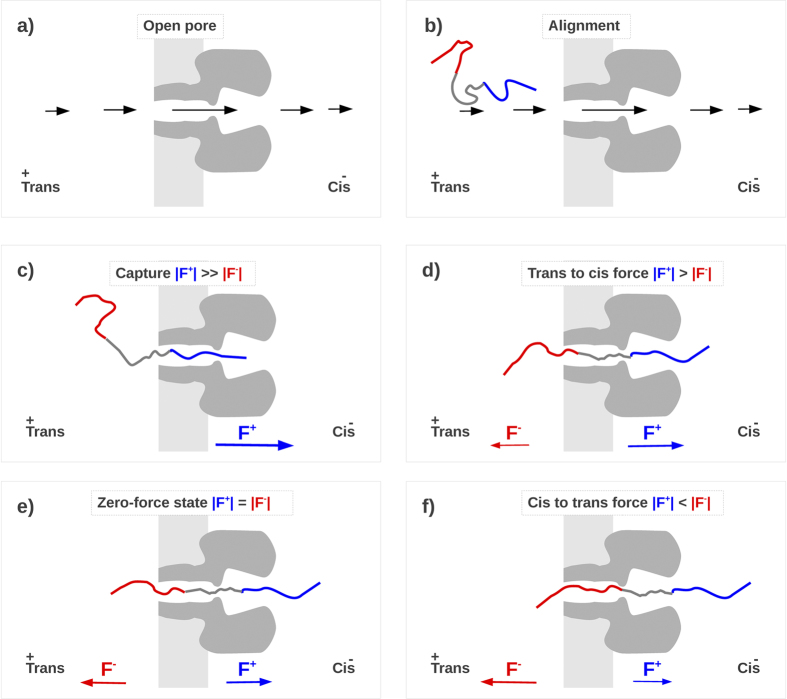
Qualitative description of the polypeptide capture and retention mechanisms. **a**) The applied voltage results in an inhomogeneous electrical field E (qualitatively sketched with black arrows) that is more intense in the pore region. **b**) This inhomogeneous field tends to align the peptide with the positive tail towards the *trans* pore mouth. **c**) The resulting electrostatic force (*F* = *q*_*effective*_ *E*, where q_effective_ represent the effective charge of the peptide moiety inside the pore), drives the polypeptide into the pore^33^,^34^. Assuming the electroosmotic effects are not significant^35^, a positive applied potential should predominately drive the positively charged end of the polypeptide into the pore. The greater the magnitude of the applied potential, the greater the chance the polypeptide will be captured. Once inside the pore, the applied potential further drives the positive end of the polypeptide into the pore. **d**) As soon as positive residues exit from the *cis* side and negative residues enters at the *trans* mouth, |F^+^| decreases while |F-| increases until the two forces approximately balance (zero net force stage, panel **e**). **f**) Further movements of the polypeptide towards the *cis* and *trans* sides (panel **d**) result in a net electrical forces that tend to drive the peptide back to the balanced force regime.

The experimental results are consistent with a simple phenomenological model illustrated in Fig. 6. We assume that the applied electric field orients the polypeptide to enter the pore (see Fig. 6b) and that there is a negligible driving force from the net electro-osmotic flow through the pore^35^. A net dielectrophoretic force acts on the polypeptide^51^, which causes the polymer’s capture and ionic current blockade (see Fig. 6c) by overcoming entropic factors. Once the polypeptide is in the pore, the electric field drives it further in (see Fig. 6d). At some point, the lagging end of the polypeptide enters the pore, and a balance between the electrostatic forces on the opposite charges at the *cis* and *trans* entrances ensues (see Fig. 6e). Thermally-induced perturbations will cause the polymer to move to the left or right (see Fig. 6f), but the driving forces will tend to restore the polypeptide to the configuration in Fig. 6e. In essence, the “zero net force” stage is a metastable state, whereby the peptide is trapped inside the α-HL pore. The peptide can escape from the pore only when thermal fluctuations experienced by it are large enough to overcome the barrier due to the combination of the applied electric field and the charges at the ends of the polypeptide. That free-energy barrier increases with the electric field **E**, hence, the larger the applied potential ΔV, the longer the mean residence time. The experimental results here do not discriminate between complete translocations or the exit of the polymer whence it entered the pore. However, for the purpose of increasing polymers in a pore for improving measurements of the molecules, translocation may not be an absolute requirement.

**Figure 7:**
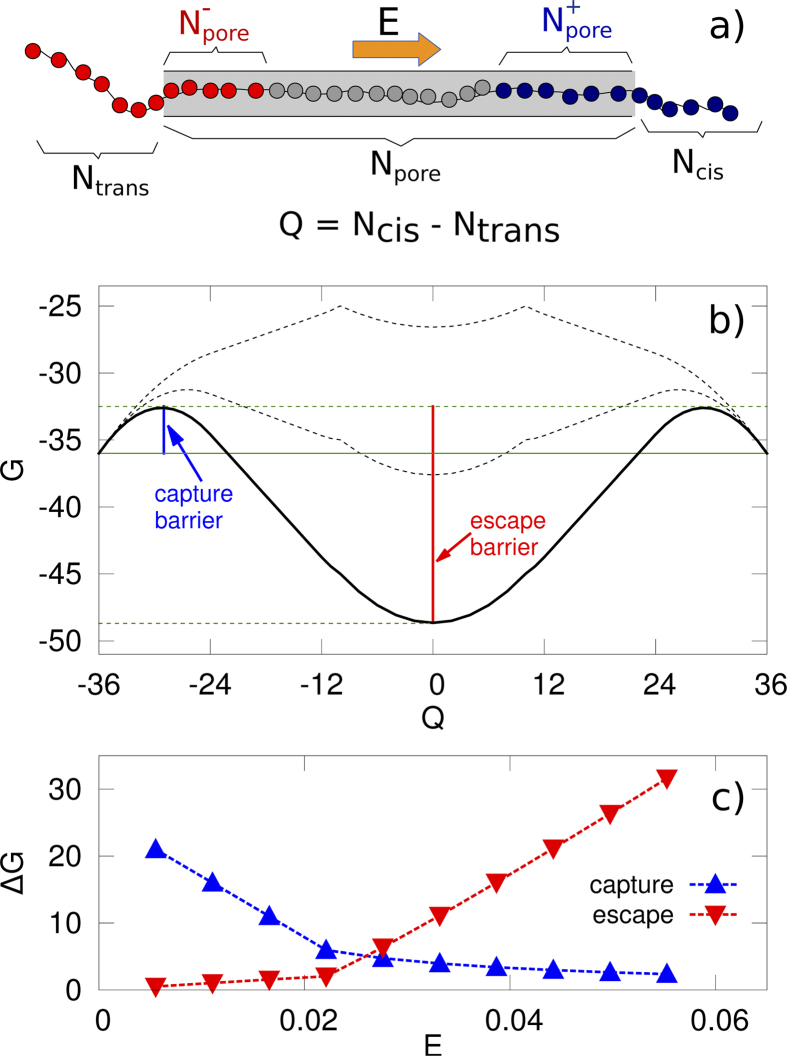
Estimates for the free-energy profile G for the polymer-nanopore interaction. The free-energy profile G as a function of the collective variable 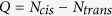 presents two local maxima and one central minimum for Q = 0 (panel **b**). The minimum is the zero net force state. The black curves refer to different values of the electric field E, the upper curve corresponding to the lower value of E. The behavior of capture and escape barrier as a function of E is reported in panel **c**. We assumed a unitary value of the dimensional constant g appearing in the equation for G_c_ (see text).

This phenomenological description can be formulated as a simple mathematical model for the free-energy profile along the translocation pathway. N_trans_ and N_cis_ are the number of residues at the *trans* and *cis* side of the pore and N_pore_ the number of residues inside the pore (see Fig. 7a). We consider only single-file motion (i.e. only linear configurations without hairpins or other secondary structures) for the portion of the polymer (a maximum of 26 residues) inside the pore. Following^29^,^52^, we describe the process using the following progress variable Q = N_cis_−N_trans_, so that Q = −36 corresponds to the initial state (N_cis_ = 0 and N_trans_  = 36) while the protein is completely translocated when Q = 36 (N_cis_ = 36 and N_trans_ = 0). A rough estimate of the shape of the free-energy G as a function of the progress variable Q, G(Q), has two contributions: a configurational contribution, G_c_(Q), and a contribution due to the external electric field, G_e_(Q). For the configurational contribution, as a first approximation, we assume the following form G_c_(Q) = -g(N_cis_(Q) + N_trans_(Q)), where g is a constant with the dimension of an energy^28^. The specific form of G_c_(Q) model is not critical here, rather the only requirement is that G_c_ is minimum when the polypeptide is not in the pore (Q = 36 or Q = −36, i.e., relatively large entropy) and it increases when the peptide enters the pore (reduction in entropy due to confinement). For the electric field contribution, G_e_(Q), we consider the work done by the external electric field **E** acting inside the pore parallel to the pore axis and directed from trans to cis side, hence
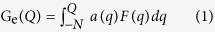
with
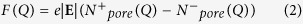


the net force acting on the peptide and a(q) the function that connects the variation in the progress variable Q to the average movement of the mass center of the peptide, in particular, a(q) = d_0_/2 when the pore is fully occupied by the peptide (N_cis _≠ 0 and N_trans _≠ 0) and a(q) = d_0_ elsewhere^29^. In equation (2) N^+^_pore_ and N^−^_pore,_ are the number of positive and negative residues inside the pore (see Fig. 7a). The expressions for N^+^_pore_, N^−^_pore,_ N_cis_ and N_trans_ can be easily derived (Supplementary Information Figure S5), and it allows to explicitly calculate F(Q), G_c_(Q) and G_e_(Q) and, consequently, the free-energy profile G(Q). As expected F(Q) = 0 for Q = 0 (polypeptide at the center of the pore), positive for Q < 0, and negative for Q > 0 (Supplementary Information Figure S5b), i.e. when the peptide moves away from the zero net force state, a net force due to the electric field **E** acts to bring it back to the stable configuration. Because F(Q) is proportional to |**E|**, for larger applied voltages is it more difficult to escape from the zero net force state. The free energy profile G(Q) is reported in Fig. 7b. In general, in order to translocate, the protein needs to pass the first free-energy barrier (capture barrier) to enter the pore. Once inside the pore, the polypeptide gets trapped in the free-energy minimum. From this minimum the protein can migrate towards both the *cis* and the *trans* side. However, in both cases an escape free-energy barrier is present. The behavior of the capture and escape barriers (see Fig. 7c) are compatible with the experimental results shown here. Indeed, by increasing E = |**E**|, the capture barrier decreases (higher probability of the peptide to get captured, and the capture rate increases with ΔV) while the escape barrier increases (lower probability to escape, 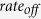 decreases with ΔV).

It is worth noting that, in the analytical model, the *cis* and *trans* escape barriers are assumed to be equal. Hence, once the peptide is captured, it is equally probable that it escapes from the *cis* or the *trans* side. In principle, the model could include qualitative information on the pore structure (e.g., parameterize the larger available volume on the vestibule side as a configurationally free-energy contribution, to consider a non-homogeneous field inside the pore^53^ or to add further energetic contributions to include interactions between the peptide and the pore). However, because we have no experimental evidence about the fraction of polypeptides that completely translocate through the pore, we limit the complexity of the model to only confirm and formalize the phenomenological representation shown in Fig. 6.

**Figure 8:**
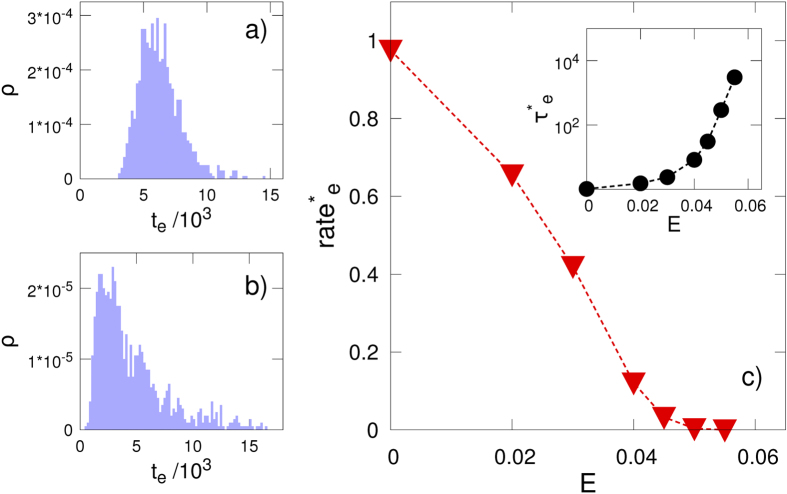
Escape time distributions from coarse-grained simulations in the **a**) absence and **b**) presence of an applied potential (E = 0.04). **c**) The dependence of the mean escape time (τ_e_) and the mean escape rate (rate_e_ = 1/τ_e_) on the electric field is shown. Both quantities are normalized with the zero electric field value, e.g. τ^*^_e_(E) = τ_e_(E)/τ_e_(0). All quantities are reported in coarse grained units, see Methods section.

A set of coarse-grained molecular dynamics simulations, which use a minimal bead-and-spring model, further support the above model. In our interpretation of the process, the current blockades are assumed to be caused by a large portion of the polypeptide in the pore. Hence, the dominant contribution to the current blockade duration is the escape from the free-energy minimum. For this reason, our simulation focuses on this part of the process. Initial configurations are prepared in the zero-net force state and the electric field E parallel to the pore axis acts only in the pore region (see Methods). The escape time t_e_, measured as the time after which the peptide has completely left the pore either from the *cis* or *trans* side, is calculated for different values of the E. It is apparent that, while the E = 0 case shows an almost symmetric distribution around the mean value, a long time tail, qualitatively in agreement with experimental results (Supplementary Information Figure S3a,c) is observed when the external electric field is present. The mean escape time τ_e_ = <t_e_>, where the average is performed over several independent simulations, and the corresponding escape rate rate_e _= 1/τ_e_, are reported in Fig. 8c. It is apparent that, as in the experimental results reported in Fig. 5a, the escape rate (rate_e_) decreases with the applied external field, reaching very small values (i.e. very long current blockades, see the inset in Fig. 8c) above a certain threshold.

As indicated above, the metastability of the peptide trapped inside the α-HL pore is determined by the net values of the electrostatic forces acting on the both ends of the polypeptide. To test this further, we use a gradient in the electrolyte concentration to alter these forces, but keep the charged state of the polypeptide invariant. This gradient leads to a change in the electrostatic potential profile near the α-HL pore entrances^22^, and cause un-balanced electric forces acting on the polypeptide while it’s in the pore, thus catalyzing its exit from the pore. Experimental results are consistent with this hypothesis (Supplementary Information Figure S6). Strictly for qualitative purposes and by neglecting to a first approximation the electroosmotic flow of water through the α-HL pore at neutral pH^21^, the dissimilarity of the electrophoretic forces acting oppositely on the peptide’s ends while the pore subjected to a salt gradient ([KCl]_cis_ < [KCl]_trans_), constitutes the main contributor to the peptide destabilization inside the pore. This was seen as a dramatic increase of the peptide transit time across the pore as compared to the case on symmetrically added salt buffers (see Supplementary Information Figure S6e).

## Discussion

By using engineered polypeptides with opposite charged groups at the N- and C- termini of an electrically neutral peptide, we demonstrate that increasing the applied potential across the pore enhances the polypeptide capture rate by the α-HL nanopore, and simultaneously increases the polymer’s residence time in the pore. The increased capture rate by the pore is a direct consequence of electrostatic interactions between the polypeptide near the pore’s *trans* entrance and the potential drop there. Once in the pore, thermal fluctuations act on the polymer, and facilitate its eventual escape from either *cis* or *trans* direction. The residence time of the polypeptide is enhanced due to an electrostatic tug of war between the charges on opposite sides of the polymer and the applied potential.

Previous attempts to increase polymer residence required the introduction of blocking groups on both ends of the molecule led to formation of a dumbbell that allowed the molecule to be arrested almost indefinitely in the nanopore^54^,^55^,^56^.

Our new method has the additional advantage of being able to perform the experiment on more than one molecule. In addition, having control over the single-molecule trap allows the interrogation of a single polypeptide interacting with the α-HL pore at tunable residence times, and possibly provide a wealth of information about the primary sequence and interaction with the pore. In another recent work, it was shown that the Coulombic interaction between a charged analyte and a metallic cluster bound to α-HL plays an important role in the residence time enhancement^57^.

In the same context of controlling biomolecule residence time in nanopores, it was shown that an electrolyte concentration gradient across the pore, with bulk salt concentrations lower on the side of biomolecule addition, can significantly enhance the capture rate without reducing the residence times^58^ A limitation of this technique occurs in particular experiments with peptides, when the low electrolyte concentration on peptide addition side hinders peptide capture by the α-HL pore’s *trans* entrance^22^ and the approach presented here overcomes these issues. Thus, because the residence time of the polypeptide in the pore can be increased with the pore bathed in a high ionic strength buffer, our approach may be particularly useful for the characterization of molecules in their native form, which can be affected via conformational changes in low ionic strength buffers. Moreover, because the polypeptide residence time in the α-HL pore is greater at larger transmembrane potentials (and not altered by high ionic strength buffers), longer (and therefore more statistically significant) measurements of the molecule can be made. This combines the intrinsically high sensitivity of the nanopore conductance during the presence of the molecule in the nanopore. The method may offer a way to probe, with improved accuracy, the internal dynamics and volumetric configuration of biomolecules, at time scales spanning hundreds of milliseconds.

Because the residence time is increased irrespective of the transmembrane potential’s polarity, the method could help control the dwell time of biomolecules in the pore, when considering the concerted action of electroosmotic flow and electrophoretic transport. Thus, during experiments in which the surface charge in nanopores and corresponding zeta potential are altered at will, calling for changes in the sign of the transmembrane potential to ensure that the electroosmotic flow counterbalances the electrophoretic transport inside the nanopore, the biomolecule braking across the pore would still occur through the mechanism described herein, regardless of the sign of the applied potential.

For analysis of polymers longer than the α-HL nanopore (~ 10 nm), it would be necessary to include elements on the primary structure of the analyte, to ensure continuous translocation of the biopolymer and enable its sequential interrogation by the nanopore. For polypeptides, we envisage at least two extensions of the present approach: (i) the attachment of distinct permanent charges (e.g., patches of charged amino acids) at one end of the polypeptide as a lead group to drive the threading of the polypeptide through the pore, while placing distinct net negative and positive amino acids at ends of segments spanning the length of the pore, to effectively tune polypeptide’s residence time, or (ii) more elaborate approaches in which a protein kinase is present on one side of the membrane that would mediate post translational, site-specific alterations in charge distribution of the polypeptide segment that visits the kinases reservoir following its threading through the pore, and therefore enhance the polypeptide’s net movement in one direction through long-range electric interactions with the applied transmembrane potential^59^.

## Methods

### Peptide synthesis

Peptides, herein termed CP2a (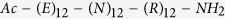) and CP2b (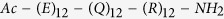), were synthesized by the solid phase method using Fmoc (9-fluorenyl-methoxycarbonyl) chemistry. Their specific length was chosen to ensure that a completely unfolded linear peptide can fit inside the ~ 10 nm thick α-HL pore (*vide infra*).

Rink Amide 4-methyl benzhydrylamine (MBHA) resin (0.30 mmol/g) was used as the support to obtain a C-terminal amidated peptide. The coupling of Fmoc-L-amino acids was performed with O-Benzotriazole-N,N,N’,N’-tetramethyl-uronium-hexafluoro-phosphate(HBTU). Amino acid side chains were protected with tert-butyl and tert-butyloxycarbonyl. Deprotection and cleavage from the resin were carried out using a mixture of trifluoroacetic acid, phenol, water, thioanisole, 1,2-ethandithiol (82.5, 5.0, 5.0, 5.0, 2.5, v/v) for 3 h at room temperature. The crude peptide was then repeatedly washed with diethylether, dried in vacuum, and purified using a preparative reversed-phase HPLC (RP-HPLC) on a Shimadzu 5-μm Shimpak ODS C18 column (20 × 250 mm). Purity of the peptide was checked by analytical RP-HPLC on a Shimpak ODS C18 column (4.6 × 250 mm). The molecular masses of the synthetic peptides were determined using the matrix-assisted laser desorption ionization MALDI-TOF mass spectrometer (Axima CFR, Kratos Analytical, Manchester, UK).

### Electrophysiology

Planar lipid membranes were obtained employing the Montal-Muller method^19^ using 1,2-diphytanoyl-sn-glycero-phosphocholine (Avanti Polar Lipids, Alabaster, AL) dissolved in n-pentane (HPLC-grade, Sigma–Aldrich, Germany). The dissolved lipid formed stable solventless bilayers across a ~120 μm in diameter orifice punctured on a 25 μm-thick Teflon film (Goodfellow, Malvern, MA), pre-treated with 1:10 hexadecane/pentane (HPLC-grade, Sigma–Aldrich, Germany), that separated the *cis* (grounded) and *trans* chambers of the recording cell. For most experiments (see text) the electrolyte used in both chambers contained 2 M KCl buffered in 10 mM HEPES, at pH = 7.3. All reagents used were of molecular biology purity. A single α-hemolysin protein pore (α-HL) (Sigma-Aldrich, Germany) was inserted in the lipid bilayer by adding ~ 0.5-2 μL from a monomeric stock solution made in 0.5 M KCl, to the grounded, *cis* chamber, under continuous stirring for about 5–10 minutes.

Once the successful membrane insertion of a single α-HL pore was attained, peptides were introduced in *trans* chamber at a bulk concentration ranging from 5 μM to 20 μM, from a 1 mM stock solution made in distilled water, and the ion current fluctuations across the α-HL pore reflecting uni-molecular reversible interactions between peptides and the α-HL protein were recorded in the voltage-clamp mode with an Axopatch 200B (Molecular Devices, U.S.A) patch-clamp amplifier. All experiments were carried out at room temperature of ~23 ^°^C. Data acquisition was performed with a NI PCI 6221, 16-bit acquisition board (National Instruments, USA) at a sampling frequency of 50 kHz, within LabVIEW 8.20 (National Instruments, USA). Before digitization, amplified electric signals were low-pass filtered at a corner frequency (f_c_) of 10 kHz. Numerical analysis and data representations were done with the help of the Origin 6 (OriginLab, USA) and pClamp 6.03 (Axon Instruments, USA) software. Data reported herein were based on three to four independent, successful experiments. When the experiments were carried out under salt gradients (see text), the transmembrane potential bias across the α-HL arising as a result of its slight anionic selectivity was offset by the voltage compensation knob on the amplifier, prior to applying the actual transmembrane voltage (ΔV)^58^. The statistical analysis on the relative blockage amplitudes induced by peptides on the electric current through a single α-HL protein, as well as the frequency and duration of the peptides-induced current blockades were analyzed within the statistics of exponentially distributed events, as previously described^5^,^21^,^22^.

### Coarse-grain simulations

The peptide is modelled as a sequence of material point (beads) of equal mass m_a_, charge q_i_, and position **r**_**i **_= (x_i_,y_i_,z_i_), with i = 1,36. The first 12 beads are positively charged (q_i_ = e, i = 1,12 with e the electron charge), while q_i_ = −e for the last 12 ones, i = 25, 36. An excluded volume potential, U(**r**_**i**_,**r**_**j**_) = 3/10 ε (**r**_**i,j**_/σ)^−12^ with **r**_**ij**_ = **r**_**i**_ – **r**_**j**_ and σ = 4.5 Å, prevents possible overlaps among non-consecutive beads, while neighboring monomers in the chain are bound with the harmonic springs potential U(**r**_**i**_,**r**_**i+1**_) = 1/2 k_c_ (|**r**_**i,i+1**_| - d_0_)^2^, with d_0_  = 3.8 Å, i.e. the typical distance between two consecutive Cα in a peptide chain, and k_c_ = 1000 ε/d_0_^2^. Electrostatic interaction among the beads are neglected; this rough approximation being quite good due the high salt concentration employed in the experiments that results in very small Debye length and, consequently, in a strong screening. The channel is modelled as a cylinder aligned with the x axis of the reference system and it acts only as a steric confinement, U_pore_(**r**_**i**_) = (V_0_(y_i_^2^ + z_i_^2^)/R_p_^2^) (1 + tanh(α x_i_(L-x_i_) ) ) with R_p_ = 4 Å, L = 100 Å, roughly corresponding to the αHL size, V_0_ = 2 ε and, α = 3 Å^−2^. The unit system employed is specified in terms of the intrinsic scales of the coarse-grained model. Specifically, lengths are given in Å, charges in electron charge e, while energy and mass are expressed as multiples m_a_ of ε. The system is coupled to a heat bath using a Langevin thermostat (gamma = 1, T = 1). The external field **E** acts along the pore axis and on the aminoacids inside the pore, x_i_ 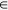 [0,L]. The resulting external force on each bead is hence **F**_**i**_ = **E**q_**i**_. As in previous works^52^,^60^,^61^ the equations of motion were integrated using a stochastic position Verlet algorithm^62^.

## Additional Information

**How to cite this article**: Asandei, A. *et al.* Placement of oppositely charged aminoacids at a polypeptide termini determines the voltage-controlled braking of polymer transport through nanometer-scale pores. *Sci. Rep.* **5**, 10419; doi: 10.1038/srep10419 (2015).

## Supplementary Material

Supporting Information
