# Supplementary material for: Placement of oppositely charged aminoacids at a polypeptide termini determines the voltage-controlled braking of polymer transport through nanometer-scale pores
Source: Sci Rep. 2015 Jun 1;5:10419. doi: 10.1038/srep10419 (PMC4450769; doi:10.1038/srep10419)
Supplement: Supporting Information [file srep10419-s1.pdf]

SUPPLEMENTARY INFORMATION FOR:

Placement of oppositely charged aminoacids at a polypeptide termini determines the voltage-controlled braking of polymer transport through nanometer-scale pores

Alina Asandei[1#], Mauro Chinappi[2#], Jong-kook Lee[3], Chang Ho Seo[3], Loredana Mereuta[4], Yoonkyung Park[5,*] and Tudor Luchian[4,*]

[1] Department of Interdisciplinary Research, Alexandru I. Cuza University, Iasi, Romania

[2] Center for Life Nano Science, Istituto Italiano di Tecnologia, Rome, Italy

[3] Department of Bioinformatics, Kongju National University, Kongju, Korea

[4] Department of Physics, Alexandru I. Cuza University, Iasi, Romania

[5] Research Center for Proteineous Materials, Chosun University, Gwangju, South Korea

[#]These authors contributed equally to this work

[*]Corresponding authors (y_k_park@Chosun.ac.kr, luchian@uaic.ro)

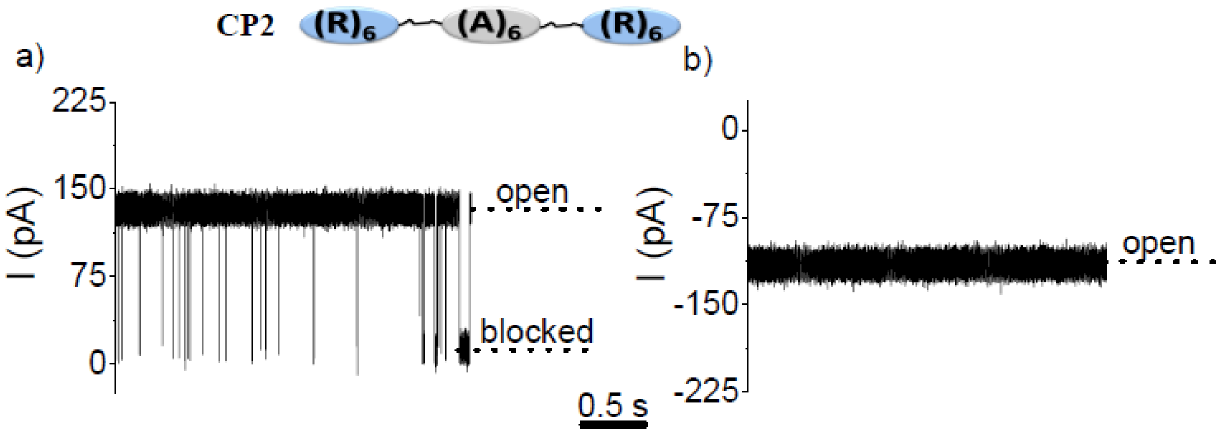

Fig. 1 SI Typical single-pore current recordings reflecting the CP2 (Ac-RRRRRRAAAAAARRRRRR-NH_2) peptide interaction with the α-HL pore immobilized in a lipid membrane, in an electrolyte containing 2 M KCl, 10 mM HEPES, pH=7.3, at ΔV=+70 mV (anel a) and ΔV=-70 mV (panel b). The bulk concentration of the peptide was 5 μM. In panel a, downward spikes reflect reduction of pore current induced by the reversible association of a peptide with an open protein pore, giving rise to the 'blocked' state, whereas the state denoted by 'open' shows level of open-pore current before a peptide partitioned within the β-barrel. At negatively applied potentials (b) the peptide does not associate and block the α-HL pore (see main text).

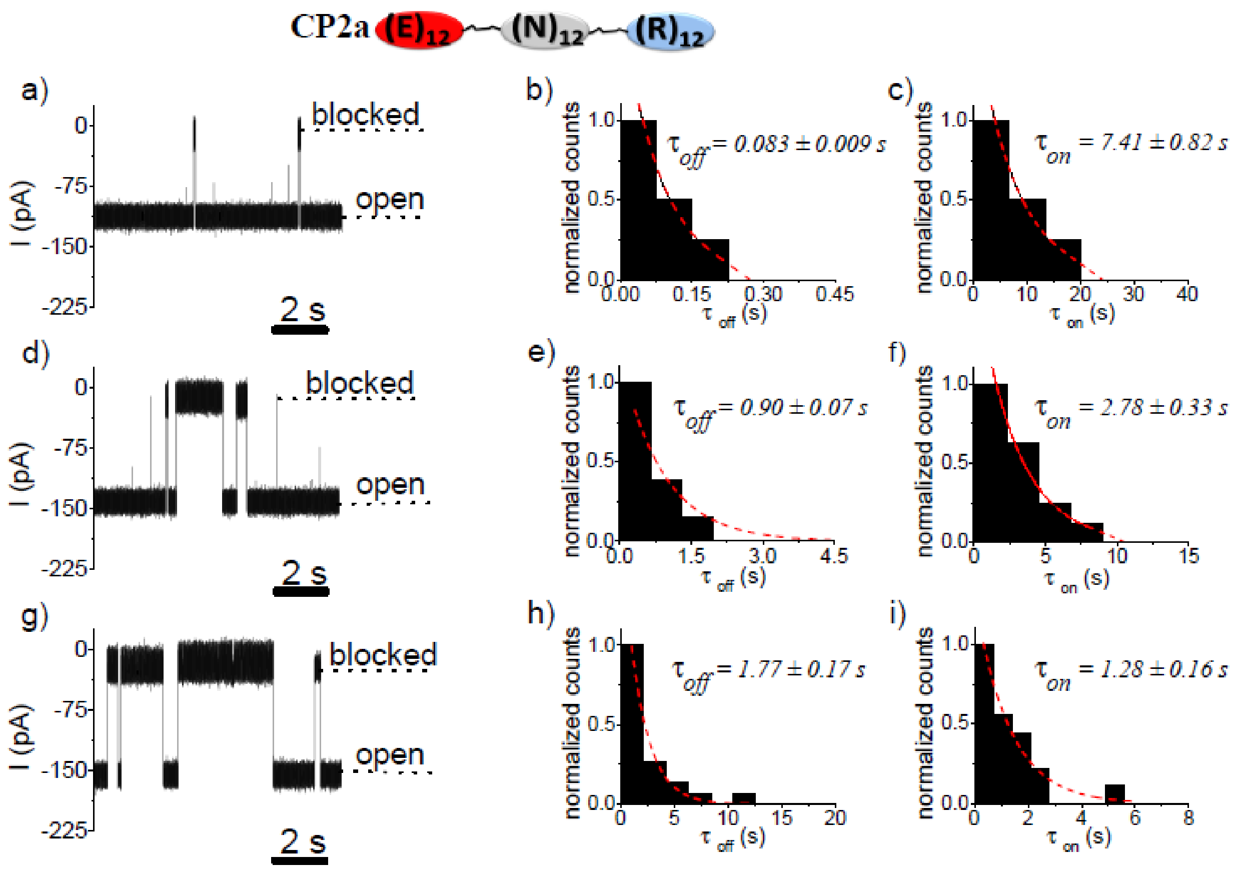

Fig. 2 SI Representative electrophysiology traces demonstrating the reversible interaction between CP2a peptides and a single α-HL pore, seen as upwardly oriented ion current changes, measured at transmembrane potentials of ΔV = -70 mV (panel a), ΔV = -90 mV (panel d) and ΔV = -100 mV (panel g). The electrolyte added symmetrically on both chambers contained 2 M KCl, 10 mM HEPES, pH=7.3 was, and the peptide was added in the *trans* chamber at a bulk concentration of 5 μM. Also shown are normalized mono-exponentially fitted distributions of blockade-events (τ_{off}) and inter-events durations (τ_{on}) characterizing the current blockades recorded at ΔV = -70 mV (panels b and c), ΔV = -90 mV (panels e and f) and ΔV = -100 mV (panels h and i).

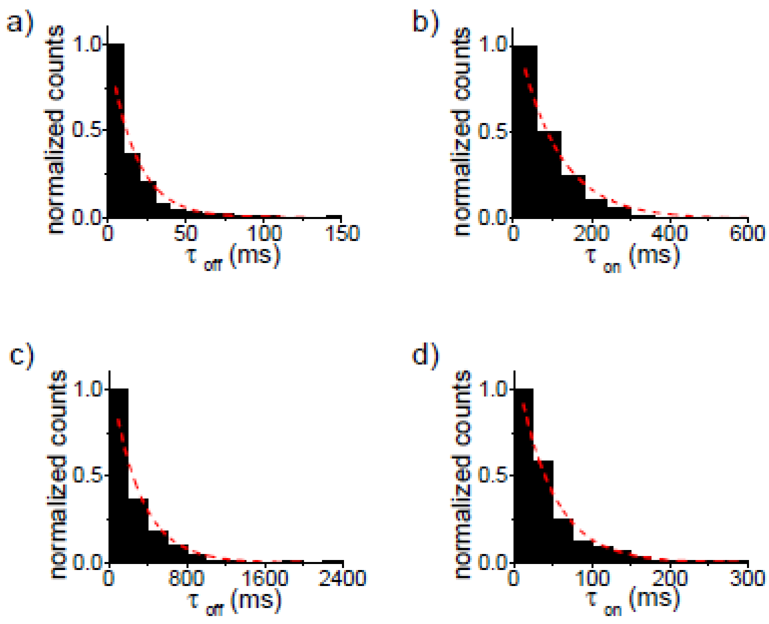

Fig. 3 SI Normalized mono- exponentially fitted distributions of blockade-events (τ_{off}) and inter-events durations (τ_{on}) characterizing the CP2a peptide-induced current blockades recorded across a single α-HL pore, clamped at ΔV = + 70 mV (panels a and b) and ΔV = + 100 mV (panels c and d) (see main text).

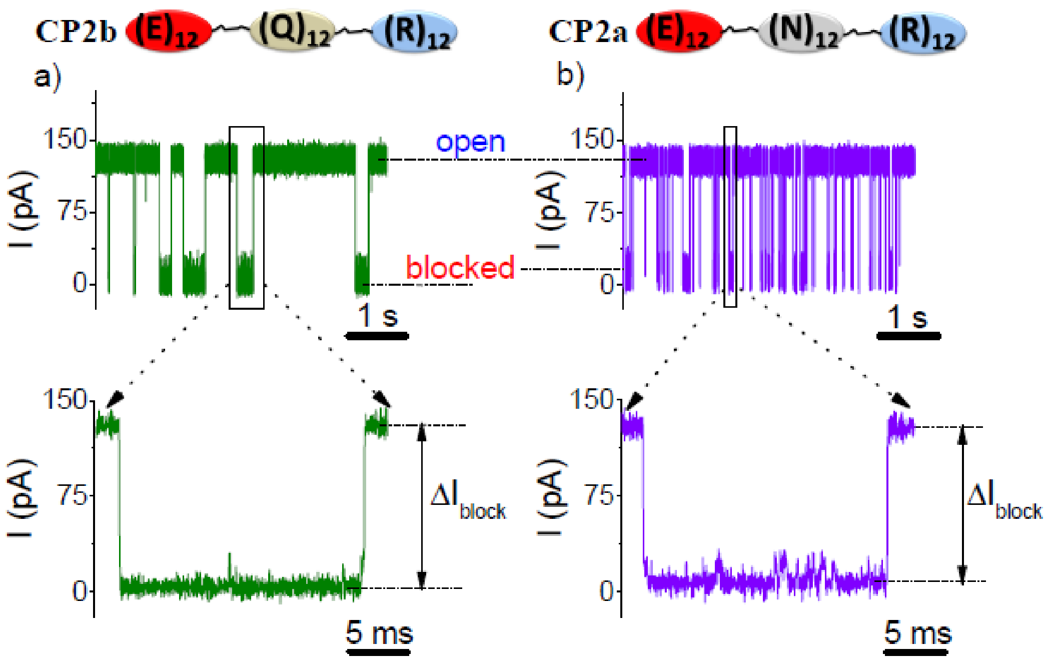

Fig. 4 SI Representative current recordings through a single α-HL pore showing the transient pore blockades by incoming CP2b (panel a) or CP2a peptides (panel b), added in the trans side at a bulk concentration of 5 µM, in an electrolyte containing 2 M KCl, 10 mM HEPES, pH=7.3, and an applied transmembrane potential ΔV = 70mV. The extent of current block by a pore-residing peptide is shown in the zoomed-in panels below (ΔI_{block} (CP2b) = -126.81 ± -0.14 pA, ΔI_{block} (CP2a) = -119.11 ± -0.31 pA).

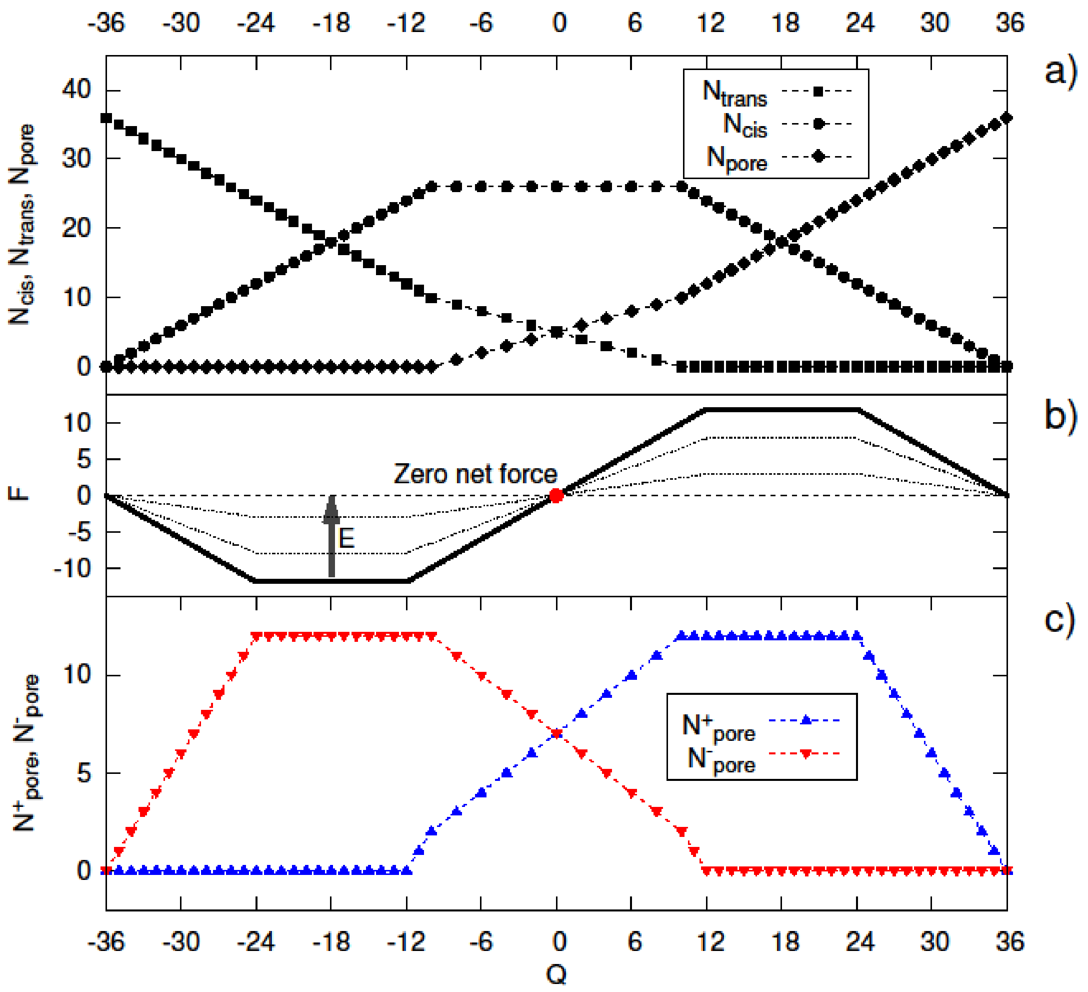

Fig. 5 SI In the analytical model presented in the text we select as progress variable $Q = N_{cis} - N_{trans}$. In the expressions for the free-energy G(Q) and the force F(Q), the quantities N^+_{pore}, N^-_{pore}, N_{cis} and N_{trans} explicitly appear. Panels a and c report these quantities as function of Q. The resulting force F(Q) is plotted in panel b for three values of E (smaller electric field correspond to lower value of the force intensity). It is apparent that for Q=0, since $N^+_{pore} = N^-_{pore}$, F(Q) = 0 (zero net force state).

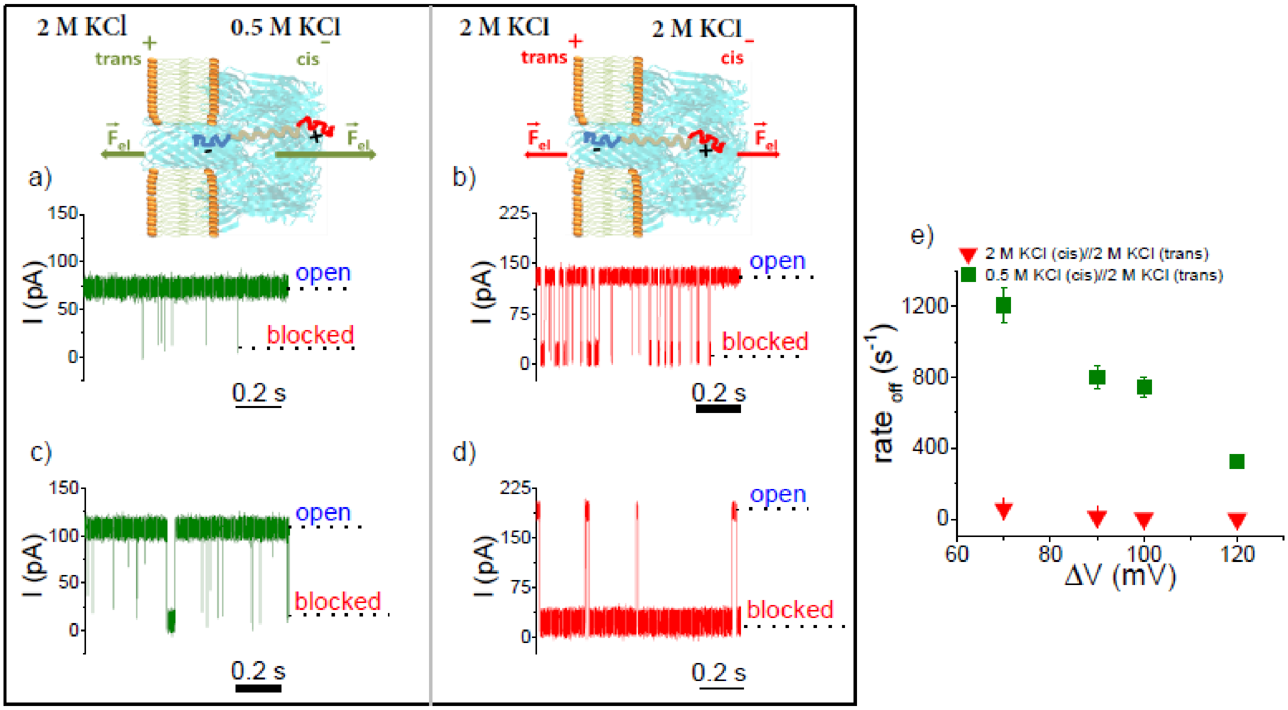

Fig. 6 SI Current blockades caused by reversible polypeptide:nanopore interactions in the presence of asymmetric and symmetric electrolyte concentrations and different applied potentials. a) and c) ΔV = + 70 mV and + 100 mV, respectively with 0.5 M KCl (*cis*) and 2 M KCl (*trans*)). b and d) ΔV = + 70 mV and + 100 mV, respectively with 2 M KCl (cis)//2 M KCl (trans). The polypeptide concentration in the bulk is 20 μM, the solution has 10 mM HEPES at pH = 7.3. e) A comparison of the calculated dissociation rates ($rate_{off}$) of the polypeptide from the nanopore for the asymmetric or symmetric solution conditions.
